# Assessment of Choroidal Vasculature and Innate Immune Cells in the Eyes of Albino and Pigmented Mice

**DOI:** 10.3390/cells11203329

**Published:** 2022-10-21

**Authors:** Ismail S. Zaitoun, Yong-Seok Song, Hammam B. Zaitoun, Christine M. Sorenson, Nader Sheibani

**Affiliations:** 1 Department of Ophthalmology and Visual Sciences, School of Medicine and Public Health, University of Wisconsin, Madison, WI 53705, USA; 2 McPherson Eye Research Institute, School of Medicine and Public Health, University of Wisconsin, Madison, WI 53705, USA; 3 Faculty of Medicine, Yarmouk University in Irbid, Irbid 21163, Jordan; 4 Department of Pediatrics, School of Medicine and Public Health, University of Wisconsin, Madison, WI 53705, USA; 5 Department of Cell and Regenerative Biology, School of Medicine and Public Health, University of Wisconsin, Madison, WI 53705, USA; 6 Department of Biomedical Engineering, University of Wisconsin, Madison, WI 53706, USA

**Keywords:** choriocapillaris, mast cells, macrophages, smooth muscle cells, uveal diseases, age-related macular degeneration

## Abstract

The visualization of choroidal vasculature and innate immune cells in the eyes of pigmented mice has been challenging due to the presence of a retinal pigment epithelium (RPE) layer separating the choroid and retina. Here, we established methods for visualizing the choroidal macrophages, mast cells, and vasculature in eyes of albino and pigmented mice using cell type-specific staining. We were able to visualize the choroidal arterial and venous systems. An arterial circle around the optic nerve was found in mice similar to the Zinn–Haller arterial circle that exists in humans and primates. Three different structural patterns of choriocapillaris were observed throughout the whole choroid: honeycomb-like, maze-like, and finger-like patterns. Choroidal mast cells were relatively few but dense around the optic nerve. Mast cell distribution in the middle and periphery was different among strains. Macrophages were found in all layers of the choroid. Thus, utilizing the simple and reliable methods described herein will allow the evaluation of transgenic and preclinical mouse models of ocular diseases that affect the choroid, including age-related macular degeneration (AMD), diabetic choroidopathy, and retinopathy of prematurity. These studies will advance our understanding of the pathophysiology, and molecular and cellular mechanisms that can be targeted therapeutically, in these diseases.

## 1. Introduction

The primary circulation system that surrounds and nourishes the outer retina and retinal pigment epithelium (RPE) is the choroidal vasculature. The choroid is a densely vascularized connective tissue consisting of three vascular layers: the anterior choriocapillaris layer that sits beneath the RPE cell basement membrane and Bruch’s membrane, the middle Sattler’s layer which is formed from medium size blood vessels, and the outermost Haller’s layer which is formed from large blood vessels [1,2]. The choriocapillaris vasculature is a distinctively anastomosed and fenestrated circulation system. The choroidal circulation is responsible for nourishing and maintaining the temperature of the photoreceptors and RPE cells [3,4,5,6]. The structural and functional integrity of the choroid is crucial for the health of photoreceptor and RPE cells, as choroidal dysfunction contributes to the pathogenesis of age-related macular degeneration (AMD) [7,8], and is associated with other diseases such as myopia [9] and diabetic retinopathy [10].

In addition to the vasculature, the choroidal stroma or connective tissue is home to different types of innate immune cells, such as mast cells and macrophages [11,12]. Mast cells are the major effector cells that play crucial roles in innate and adapted immunity along with autoimmunity. The activation of mast cells induces their degranulation which results in the release of different chemokines, cytokines, histamine, and proteases [13]. In turn, released proteases, such as tryptase, chymase, and cathepsin G, among others, cause connective tissue and basement membrane degradation. Mast cells also produce proangiogenic factors, including the vascular endothelial growth factor (VEGF) [14] that initiates or modulates angiogenesis. Recent evidence suggested important roles played by choroidal mast cells in the development of AMD [15]. Macrophages normally exist in the whole choroidal connective tissue. During their resting state, macrophages sense their surroundings and interact with choroidal vasculature and RPE cells [16]. Macrophages affect vascular development and homeostasis. Clinical and preclinical studies demonstrated that changes in the normal state of choroidal macrophages are associated with vascular degeneration during aging and in patients with AMD [16,17,18].

Our understanding of the causes of AMD and other uveal diseases requires further delineation. One main stumbling block is the lack of simple and reliable methods to visualize blood vessels and immune cells in wholemount choroid of both pigmented and albino mice using immunostaining. Here we report a reproducible method to visualize the choroidal blood vessels, mast cells, and macrophages, independently or simultaneously, in albino mice. When combined with melanin bleaching, these methods can be used on pigmented mice. Using these methods, here we visualized and assessed the blood vessels, mast cells, and macrophages in the choroid of three albino (FVBN, CD-1, BALB/cJ) and one pigmented (C57BL/6J) mouse strains.

## 2. Materials and Methods

### 2.1. Animals

Six-week-old animals of both sexes from FVBN, CD-1, BALB/cJ, and C57BL/6J mice were used for these studies. CD-1 mice were from Charles River Laboratories (Wilmington, MA, USA). FVBN, BALB/cJ, and C57BL/6J were obtained from Jackson Laboratory (Bar Harbor, MA, USA) and were bred in our mouse colony at UW-Madison. Eyes from a comparable number of males and females were used in these studies. Mice were housed and allowed *ad libitum* access to standard rodent chow and water. Animals were euthanatized via exposure to carbon dioxide.

### 2.2. Tissue Preparation for Staining

Eyes were enucleated, washed in phosphate-buffered saline (PBS), and fixed in 4% paraformaldehyde (PFA: Cat# 15710; Electron Microscopy Sciences, Hatfield, PA, USA) for 1 h on a rocker at room temperature. Eyes were then washed three times in PBS. The eyeball was first cleaned from all muscle, fat, and conjunctiva tissues attached to the sclera. To attain only the choroidoscleral structure, the cornea, lens, and retina were dissected away. Care was taken to keep the limbus attached to the choroidoscleral structure. This tissue was then kept for 1 h in the blocking solution (1% BSA, 0.3% Triton X-100, 0.05% Azide, all in 1X PBS) at room temperature on a rocker.

### 2.3. Fluorescence Immunostaining of Choroidal Blood Vessels and Macrophages

The antibodies were prepared in the blocking solution (1% BSA, 0.3% Triton-X100, 0.05% sodium azide, all in PBS) and kept on ice. The goat anti-podocalyxin (R&D Systems, Minneapolis, MN, USA; Cat# AF1556) was used to stain the choroidal vasculature by diluting 1 μL of antibody in 500 μL of blocking solution. The rabbit anti-Iba1 (Wako Pure Chemical Industries, Osaka, Japan; Cat#: 019-19741) was used to stain choroidal macrophages by diluting 1 μL of antibody in 500 μL of blocking solution. Once antibodies were prepared, the blocking solution was removed away from the tissue and incubated with antibody solutions on a rocker in the cold room for 2–3 days. The tissues were then washed with PBS (3 times, 5 min each) at room temperature on a rocker. The appropriate secondary antibodies were prepared by diluting 1 μL of each secondary antibody in 500 μL of blocking solution and were added to the tissues. Secondary antibodies were from Jackson ImmunoResearch Laboratories (West Grove, PA, USA) including donkey anti-goat-Cy5 (Cat#: 705-175-147) and donkey anti-rabbit-Cy2 (Cat#: 711-225-152). Samples were incubated overnight in the secondary antibody solution on a rocker at 4 °C. The stained choroidoscleral structures were flattened by several radial cuts and mounted flat with an RPE layer facing upward on a slide with DAPI Fluoromount-G (Southern Biotech, Birmingham, AL, USA; Cat#: 0100-20). Signals were visualized by fluorescence microscopy and images were taken in digital format using Nikon confocal microscope system A1+. Captured images were then processed using NIS elements software (Nikon, Japan).

### 2.4. Fluorescence Immunostaining of Choroidal Arteries

The eyeballs from 3-week-old FVBN mice were enucleated, washed in PBS, and fixed in 4% PFA for 1 h at room temperature on a rocker, and then washed three times in PBS. The choroidoscleral structure was dissected and kept for 1 h in 1 mL of the blocking solution (1% BSA, 0.3% Triton X100, 0.05% Azide, all in PBS) at room temperature on a rocker. The tissue was incubated with anti-α-SMA-FITC (Sigma, #F3777, 1:500 in the blocking solution) overnight in the cold room on a rocker. The choroidoscleral structures were then washed in PBS, flat-mounted, and imaged as described above.

### 2.5. Fluorescence Staining of Choroidal Mast Cells

Heparin is a mixture of sulfated glycosaminoglycan that exists in mast cells. Avidin is a tetrameric glycoprotein that binds to heparin. Fluorescence-conjugated avidin is successfully used to visualize mast cells in both humans and mice [19,20,21,22,23]. We used rhodamine-avidin (Vector Laboratories, Burlingame, CA, USA; Cat#: A-2012) to stain the mast cells. Rhodamine-avidin was diluted (1:500) in the blocking buffer and samples were kept in the cold room on a rocker overnight. For albino mice, tissues were washed three times in PBS and mounted on a slide. When co-staining of mast cells and blood vessels or macrophages was desired, rhodamine-avidin was added to the secondary antibodies mix.

In the case of pigmented eyes, bleaching in hydrogen peroxide was necessary. H_2_O_2_ has been used by others to bleach melanin from pigmented eyes with the purpose of imaging the intact retina or the subretina [24,25]. To make sure hydrogen peroxide bleaches melanin without affecting heparin integrity in mast cells from C57BL/6J mice, choroidoscleral structures were crosslinked using Sulfo-SMCC (sulfosuccinimidyl 4-(N-maleimidomethyl) cyclohexane-1-carboxylate) (ThermoFisher, A39268). Sulfo-SMCC is an amine-to-sulfhydryl crosslinker and it was used here to stabilize heparin. To ensure successful crosslinking, each choroidoscleral tissue was incubated in 100 μL of Sulfo-SMCC in a 1.5 mL Eppendorf tube for 12–24 h in the cold room on a rocker. Tissues were washed in PBS (3 times, 5 min each) at room temperature on a rocker before fixing the tissue in 4% PFA for 1 h at room temperature on a rocker.

### 2.6. Bleaching and Redeveloping the Fluorescence Signal

For labeling the choroidoscleral structure from pigmented mice, the same staining steps described above were performed first. Extra steps were then performed, including post-stain fixation and bleaching. When Avidin staining was done, the stained tissue was kept in Sulfo-SMCC for 12–24 h in the cold room on a rocker as detailed above. Tissues were washed with PBS (3 times, 5 min each) at room temperature on a rocker, and then fixed in 4% PFA for 1 h at room temperature on a rocker. Fixed tissues were washed with PBS (3 times, 5 min each) on a rocker at room temperature before bleaching. For bleaching, we used 1% H_2_O_2_ (in 1xPBS) to maintain the natural epitope and tissue architecture. Tissues were submerged in 1% H_2_O_2_ and kept in a 55 °C water bath for 4–6 h. The bleaching solution was changed two to three times with warmed fresh 1% H_2_O_2_ in 1xPBS. Bleaching was completed once the tissue became relatively transparent. The 1% H_2_O_2_ solution was removed, the tissue was thoroughly washed with PBS (3 times), and then incubated with the 1.5 mL of blocking solution in a 2 mL tube. For re-staining of the bleached tissues, secondary antibodies (and if needed the rhodamine-avidin), prepared as described above, were added to the bleached tissues, and kept overnight in the cold room on a rocker. Tissues were then washed three times in PBS, mounted, visualized, and imaged as described above. To make sure bleaching does not compromise the sample’s structural features, choroidoscleral tissues from albino FVBN mice were stained with an anti-podocalyxin antibody and imaged before and after bleaching. With bleaching, no noticeable changes to the choroidal vasculature were noted, thus demonstrating the feasibility of bleaching choroidoscleral tissues with 1% H_2_O_2_ to study the choroidal vasculature and other cells in the pigmented mice.

### 2.7. Statistical Analysis

Mast cells were manually counted. To evaluate statistical differences in multiple groups, a one-way ANOVA followed by Tukey’s multiple comparison test was performed. Results are presented as mean ± SD. *p* < 0.05 was considered significant. GraphPad Prism 8.0 (GraphPad Software, San Diego, CA, USA) was used to perform statistical analysis and create graphs.

## 3. Results

The ocular choroidal tissue contains blood vessels and stroma (extravascular tissue). Choroidal blood vessels are composed of arteries, arterioles, veins, venules, and a capillary bed, namely choriocapillaris. Blood vessels in the choroid become smaller as they branch while descending from the sclera border towards the Bruch’s membrane. This branching pattern allows the distinction of three vascular layers in humans: the Haller’s layer of the large vessels close to the sclera, the Sattler’s layer of medium size blood vessels, and the choriocapillaris layer of highly anastomosed capillary bed next to Bruch’s membrane. Although on a much smaller scale because of eye size, we think our data show that the three layers are discernible in mice as well (Supplementary Video S1). The stroma is a connective tissue that encompasses the blood vessels and harbors immune cells, including mast cells and macrophages, neural tissues, and other types of cells. Choriocapillaris and Bruch’s membrane are fused together via a basement membrane and the intercapillary pillars (also called septa), which are a part of the choroidal connective tissue.

### 3.1. Visualization of the Blood Vessels in the Choroid

Eyes from the albino FVBN mice were used to screen for blood vessel markers normally used to label the vasculature in the retina and in other tissues. We searched for markers that, according to our criteria, would robustly and exclusively label all blood vessels, but nothing else in the choroid. Anti-podocalyxin antibody was the only antibody that met these criteria. Anti-α-SMA antibody robustly labeled smooth muscle cells in the choroid. The following markers failed to meet the criteria for vascular staining: *Griffonia simplicifolia isolectin* B4 (IB4) and antibodies for intercellular adhesion molecule 2 (ICAM2), type I collagen, and type IV collagen. These markers either did not label the choroidal vasculature in our hands at all, or they labeled only some large blood vessels. The use of anti-α-SMA and anti-podocalyxin together allowed the clear visualization of the arterial system, venous system, and choriocapillaris in the mouse choroid.

### 3.2. Arterial System

**Figure 1:**
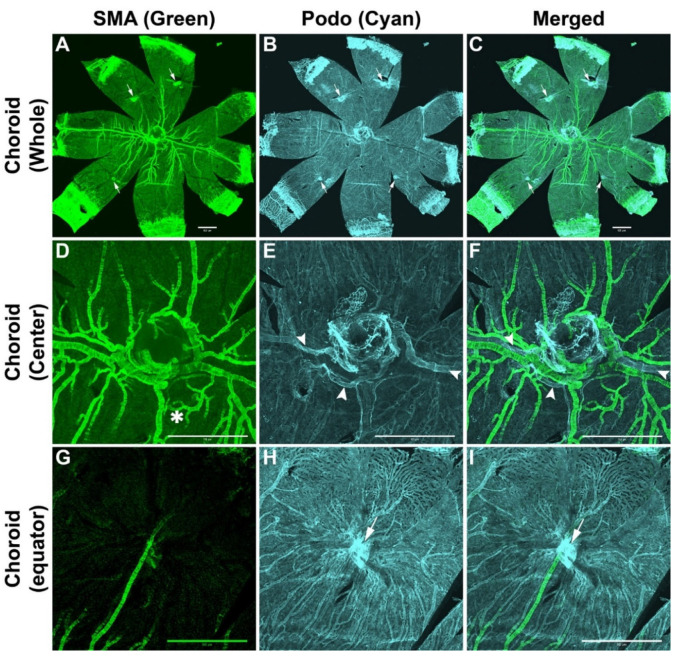
Visualization of the arterial and venous systems of the mouse choroid. (**A**,**D**,**G**) The anti-α-SMA staining labels the major arterial system in the choroid. The two long posterior ciliary arteries (LPCAs) extend nasally and temporally. It is obvious that LPCAs feed the entire choroidal vasculature. LPCAs send branches around the optic disc as it appears from the α-SMA staining, forming a vascular circle, such as the Zinn–Haller arterial circle in the choroids of humans and primates. Secondary arteries sometimes form loops (**D**, asterisk). At least two major LPCA-derived arteries feed the superior and inferior parts of the choroid. LPCAs and their major LPCA-derived arteries run perpendicular to each other, dividing the choroid into four quadrants. (**B**,**E**,**H**) Anti-podocalyxin staining strongly labels most of the choroidal vasculature except the arterial system, especially in the center of the choroid. Four vortex veins are observed at the equator of the choroid (arrows). A second venous system can be seen in the center of the choroid (arrowheads). These central veins appear to drain blood from the center of the choroid. (**C**,**F**,**I**) Merged images from staining with anti-α-SMA and anti-podocalyxin antibodies show the detailed structure of all choroidal components, including arteries, veins, and choriocapillaris. Thus, both venous systems in the choroid are more easily identified with respect to the rest of the vasculature, especially arteries. Number of choroids examined = 6. All representative images were from a 3-week-old FVBN mouse and captured using a confocal microscope. Scale bars: 500 μm.

The anti-α-SMA staining labels the major arterial system in the choroid. When combined with podocalyxin staining, the double staining allowed the clear visualization of the major arterial system with respect to the rest of the choroidal vasculature (Figure 1A–I). The posterior ciliary artery (PCA), which branches from the ophthalmic artery, is the major artery that feeds the retina and choroid. Once in the center of the choroid, close to the optic disc, the PCA branches into two long posterior ciliary arteries (LPCAs) that extend nasally and temporally. These LPCAs feed the entire choroidal vasculature and the blood vessel systems in the iris and ciliary body. Right after they enter the iris, the LPCAs give rise to two symmetrical branches forming the circular iris artery. Both nasal and temporal LPCAs send branches around the optic disc, as it appears from the α-SMA staining, forming a vascular circle (Figure 1A–F), similar to the Zinn–Haller arterial circle in the choroids of humans and primates. At least two major LPCA-derived arteries feed the superior and inferior parts of the choroid. LPCAs and their major LPCA-derived arteries run perpendicular to each other, dividing the choroid into four quadrants. From the peripapillary area to the equator, the LPCAs give rise to two to four branches on each side, which feed the associated choriocapillaris. The closer the branch is to the peripapillary area, the larger it is in circumference and length. These branches run parallel to their parent LPCA while branching themselves away from the LPCAs, but they stay in the same plane before these arterioles change direction directly and connect with the choriocapillaris. The major arteries that feed the superior and inferior parts of the choroid branch forward and in all directions. Except for LPCAs, only a few if any major arteries or arterioles exist in the periphery of the choroid. We were unable to identify short posterior ciliary arteries (SPCAs) in the mouse choroids we examined in this study. SPCAs are known to exist in the human choroid.

### 3.3. Venous System

Podocalyxin staining labeled all the choroidal vasculature well with the exception of the LPCAs along with their major branches in the peripapillary area, which were faintly labeled. Interestingly, these faintly podocalyxin-labeled blood vessels are the blood vessels that are labeled with α-SMA. Double staining with α-SMA and podocalyxin allowed clear visualization of major drainage systems with respect to the rest of the choroidal vasculature (Figure 1A–I). Two major drainage systems could be identified: vortex veins, which drain blood from the iris, the periphery of the choroid, and the portion of the posterior choroid close to the equator. There are four vortex veins close to the equator of the choroid on the dorsal, ventral, nasal, and temporal sides (Figure 1A–C,G–I). For each vortex vein, several large veins converge together to form larger sacs of blood vessels before these sacs come together to form the vortex vein. The upper part of the vortex veins, which is closer to the sclera, was sometimes positive for α-SMA staining (Figure 1A,D). In addition to the vortex veins, we identified veins in the center of the choroid that appear to be the peripapillary vein connecting to the posterior ciliary vein. This venous system drains blood vessels from the center of the choroid (Figure 1E,F).

### 3.4. Choriocapillaris

**Figure 2:**
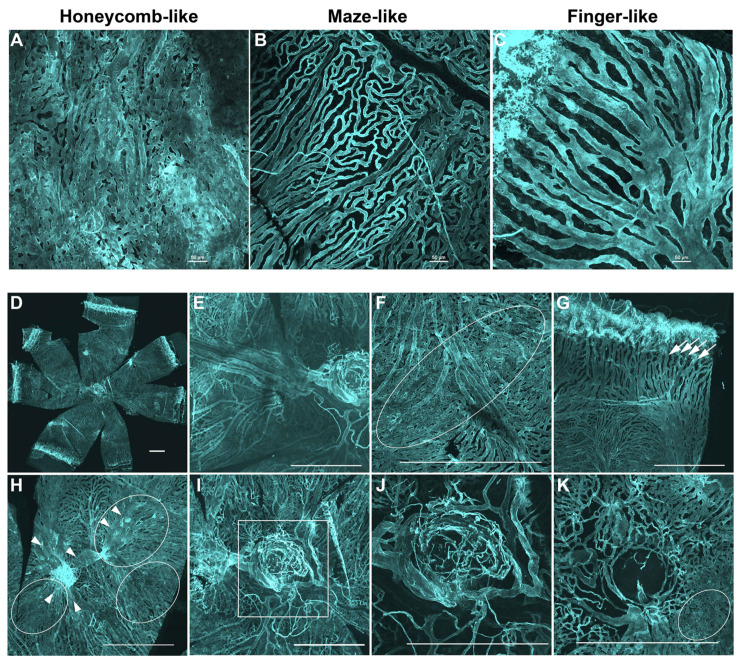
Visualization of the choriocapillaris and veins in the mouse choroid. The choriocapillaris display three different patterns, including the honeycomb-like pattern (**A**), the maze-like pattern (**B**), and the finger-like projections pattern (**C**,**D**). Low magnification image of the whole choroidal vasculature of 6-week-old FVBN mouse. (**E**) High magnification of choroid vasculature that includes the optic disc, the posterior pole, and part of the equator. Other high-magnification images of the choroid show the choriocapillaris in the equator away from the vortex veins (**F**), periphery (**G**) and equator with the vortex vein (**H**), and optic disc area (**I**–**K**). Inset in **I** was focally dissected to separate the large blood vessels (**J**) from the choriocapillaris (**K**). The honeycomb-like pattern observed in the middle of the equator, in the vicinity of the tributaries of the vortex vein, and on one side of the optic disc (**F**,**H**,**K**; oval shapes). Please note the tributaries of the vortex vein (**H**; arrowheads). The finger-like projections pattern was observed in the periphery (**G**; arrows). The maze-like pattern was observed in the equator (**F**), mid-periphery (**G**), and in the peripapillary areas (**K**). Tissues were stained using anti-podocalyxin antibody. Number of choroids examined = 14. All representative images were from 6-week-old FVBN mice and captured using confocal microscopy. Scale bars: 50 (**A**–**C**) and 500 μm (**D**–**K**).

The choriocapillaris shows different structural patterns in different locations of the choroid (Figure 2A–K). Generally, three different patterns can be found: a dense honeycomb-like pattern (Figure 2A), a maze-like pattern (Figure 2B), and a finger-like projection pattern (Figure 2C). The finger-like projection pattern with large intercapillary spaces (septa) exists mainly in the far periphery of the choroid close to the anterior segment (Figure 2G). These finger-like vessels form arcades right before connecting with blood vessels of the ciliary body at the ora serrata. The other two patterns can be found in different parts of the choroid. We found the honeycomb pattern, with small septa, in a small strip in the equator of the choroid (Figure 2F), in the vicinity of the tributaries of the vortex vein (Figure 2H) and close to the optic disc (Figure 2K). The maze-like pattern is dominant in the mid periphery, in the equator, and in the peripapillary areas, where their septa area is large, and varies a lot (Figure 2F–K). The caliber of the capillaries varies as well. We found the choriocapillaris of 6-week-old FVBN mice to be light in nature where the septa area is considerably large and the dense choriocapillaris is uncommon. Due to this finding, we next examined whether other mice strains of similar age (6-week-old) have similar choriocapillaris density. We investigated the choroid from two more strains of albino mice, including BALB/cJ and CD-1, and from the pigmented C57BL/6J mice.

### 3.5. Choriocapillaris from Albino and Pigmented Mice

**Figure 3:**
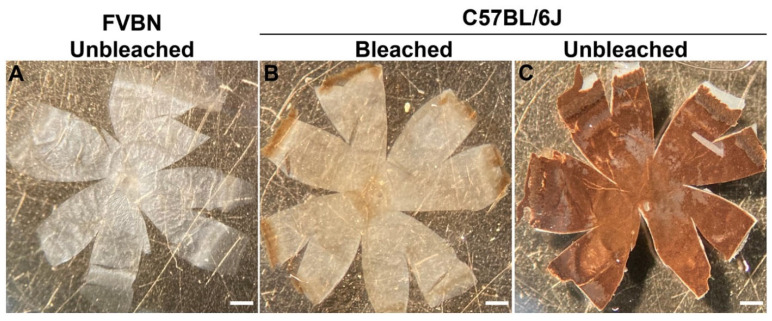
Whole choroidoscleral tissue from albino (FVBN, unbleached) (**A**), bleached C57BL/6J (**B**), and unbleached C57BL/6J (**C**) mice. Exposing the choroidoscleral tissue from pigmented C57BL/6J mice to 1% H_2_O_2_ for 4–5 h at 55 °C in a water bath was sufficient to bleach almost all melanin in the tissue, as it becomes almost transparent. Representative images were from 6-week-old FVBN and C57BL/6J mice and captured in digital format. Scale bars: 500 μm.

To examine the choroid and its cellular components from the pigmented C57BL/6J mice, we first needed to eliminate the melanin pigment that exists in the RPE and choroidal melanocytes. The goal was to bleach the melanin without damaging the tissue. For this purpose, we successfully bleached melanin in the eye in 1% hydrogen peroxide (1% H_2_O_2_). H_2_O_2_ has been used by others to bleach melanin from pigmented eyes with the purpose of imaging the intact retina or the subretina [24,25]. Figure 3 shows choroidoscleral flat-mounts from albino FVBN, bleached C57BL/6J, and from unbleached C57BL/6J mice. After successfully developing these novel methods for staining the choroidal vasculature and clearing the pigment from the eye, we were able to compare the choroidal vascular structure from both albino and pigmented mice for the first time.

**Figure 4:**
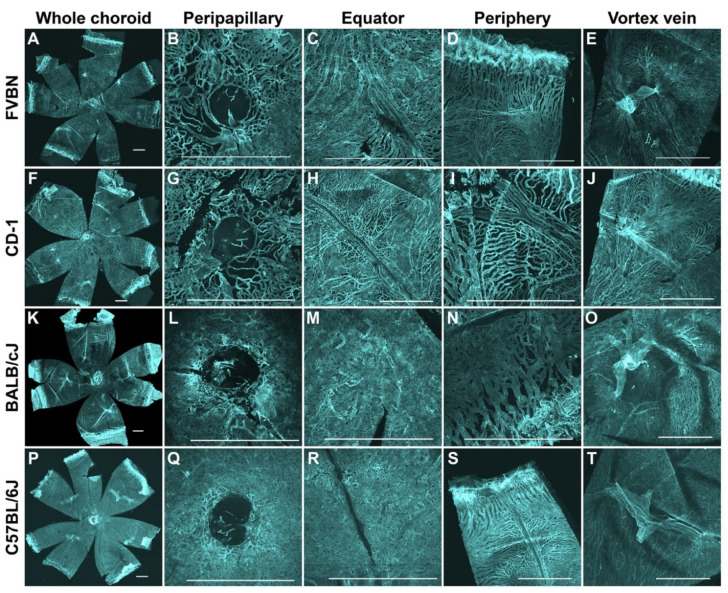
Visualization of the choroidal vasculature from albino and pigmented mice. Representative images from FVBN (**A**–**E**), CD-1 (**F**–**J**), BALB/cJ (**K**–**O**), and bleached C57BL/6J (**P**–**T**) mice. Shown are representative images from the low magnification of the whole choroidal vasculature, along with high magnification from the peripapillary, equator, periphery, and vortex vein. The choriocapillaris displays three different patterns, including finger-like projections, honeycomb-like, and maze-like patterns. Images show that the choroids from FVBN and CD-1 albino mice are sparse and less dense as compared with choroids from BALB/cJ albino mice. Choriocapillaris of the C57BL/6J mice is similar to that in the BALB/cJ mice, but slightly denser. Tissues were stained using an anti-podocalyxin antibody. Number of choroids examined = 14 (FVBN), 7 (CD-1), 9 (BALB/cJ), and 9 (C57BL/6J). All representative images were from 6-week-old animals and captured using a confocal microscope. Scale bars: 500 μm.

Figure 4 shows representative images of the choroidal vasculature from 6-week-old FVBN, CD-1, BALB/cJ, and C57BL/6J mice. The whole choroid–sclera and the vortex vein are shown. Choriocapillaris from the peripapillary, equator, and periphery regions are shown as well. The choroidal structure from the FVBN is shown in Figure 2 and Figure 4A–E and was described above. In the CD-1 mice, the maze-like pattern is dominant in the peripapillary, equator, and in periphery around the LPCAs (Figure 4F–J). The finger-like and arcade structures exist in the far periphery of the choriocapillaris (Figure 4I). The honeycomb pattern was rarely found in the peripapillary, but it was seen close to the vortex vein (Figure 4J). In the BALB/cJ mice, the honeycomb pattern exists in the peripapillary, equator, and vortex vein areas of the choriocapillaris (Figure 4L,M). The maze-like pattern exists in the equator and mid-periphery areas (Figure 4M,N). The finger-like and arcade structures exist in the far periphery of the choriocapillaris (Figure 4N). The dense nature of the choriocapillaris in BALB/cJ mice is obvious. In the C57BL/6J mice, the honeycomb pattern exists in the peripapillary, the equator areas, and around the vortex vein of the choriocapillaris (Figure 4Q,R,T). The maze-like pattern exists in the equator and mid-periphery areas (Figure 4R,S). The finger-like and arcade structures exist in the far periphery of the choriocapillaris (Figure 4S). The dense nature of the choriocapillaris in this strain is clear. Taken together, the choriocapillaris from CD-1 mice seemed similar to that from FVBN mice. These two albino strains showed choriocapillaris atrophy, at least at this age (6-week-old), where capillary calibers are small and their intercapillary spaces are commonly large. This is in contrast with choriocapillaris from the albino BALB/cJ and pigmented C57BL/6J mice. In the C57BL/6J mice, the choriocapillaris is similar to that in the BALB/cJ mice, but slightly denser.

### 3.6. Visualization of Mast Cells in the Choroid of Albino and Pigmented Mice

**Figure 5:**
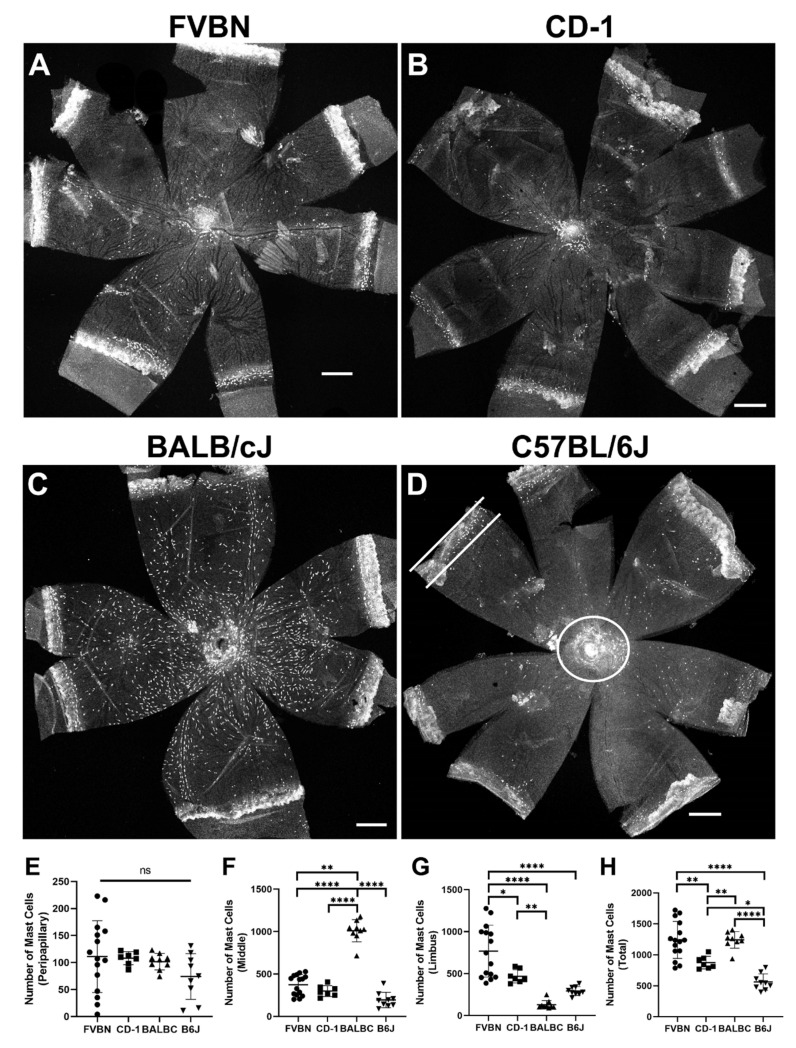
Visualization of mast cells in the choroid of albino and pigmented mice. Representative images showing the distribution of mast cells in choroidoscleral tissues from FVBN (**A**), CD-1 (**B**), BALB/cJ (**C**), and bleached C57BL/6J (**D**) mice. The counts of mast cells in the peripapillary (**E**), middle (**F**), limbus (**G**), and whole (**H**) choroid of the four examined strains of mice were determined and statistically analyzed. Each data point represents one choroid. A small number of mast cells exist around the optic disc area (**D**; white circle), but the majority of mast cells reside in the limbus (**D**; flanked by two white lines). Mast cells showed similar distribution pattern in the choroidoscleral tissue from C57BL/6J, CD-1, and FVBN mice. While a small number of mast cells exist around the optic disc area in these three strains of mice, the majority of mast cells reside in the limbus. An approximately similar number to those mast cells in the limbus is distributed in the rest of the choroid (referred to here as middle). BALB/cJ mice showed a unique mast cell distribution as compared to the other three examined strains in this study. Mast cells in BALB/cJ appear larger in size and were approximately equally distributed throughout the whole choroidal tissue. Mast cell counts in these four strains showed BALB/cJ and FVBN to have similar highest numbers. C57BL/6J mice had the lowest number. Rhodamine-avidin was used to label mast cells in the choroid. All representative images were from 6-week-old animals and captured using confocal microscopy. Number of choroids examined = 14 (FVBN), 7 (CD-1), 9 (BALB/cJ), and 9 (C57BL/6J). Scale bars: 500 μm. Results are presented as mean ± SD. ns: not significant, * *p* < 0.05, ** *p* < 0.01, and **** *p* < 0.0001.

To visualize mast cells in the choroid from albino and pigmented mice, we used rhodamine-avidin. Avidin recognizes and binds heparin in mast cells, and fluorescence conjugated avidin is successfully used to visualize mast cells in both human and mice [19,20,21,22,23]. Heparin is a mixture of sulfated glycosaminoglycan. To make sure hydrogen peroxide bleached melanin without affecting heparin integrity in mast cells from C57BL/6J mice, choroidoscleral structures were crosslinked with Sulfo-SMCC before bleaching. Sulfo-SMCC is an amine-to-sulfhydryl crosslinker (please refer to Methods section for details). Figure 5 shows mast cell distribution in choroidoscleral flat-mounts from FVBN (Figure 5A), CD-1(Figure 5B), BALB/cJ (Figure 5C), and C57BL/6J (Figure 5D) mice. Mast cells in these four mouse stains were counted, analyzed, and graphed (Figure 5E–H). Mast cells reside in the vicinity of large blood vessels. Their distribution and density varied among different strains. Mast cells showed a similar distribution pattern in the choroidoscleral tissue from C57BL/6J, CD-1, and FVBN mice. In these three stains, a small but concentrated number of mast cells exist around the optic disc area, but the majority of mast cells reside in the limbus. A similar number to those mast cells in the limbus is distributed throughout the rest of the choroid (referred to here as the middle choroid). Mast cell distribution in the BALB/cJ is unique, where mast cells are distributed equally throughout the whole choroidoscleral tissue. In addition, mast cells in this line of mice appear to be larger in size. Mast cell counts in all four examined strains showed BALB/cJ and FVBN to have the highest number of mast cells. C57BL/6J mice had the lowest number of mast cells.

### 3.7. Visualization of Macrophages in the Choroid of Pigmented Mice

**Figure 6:**
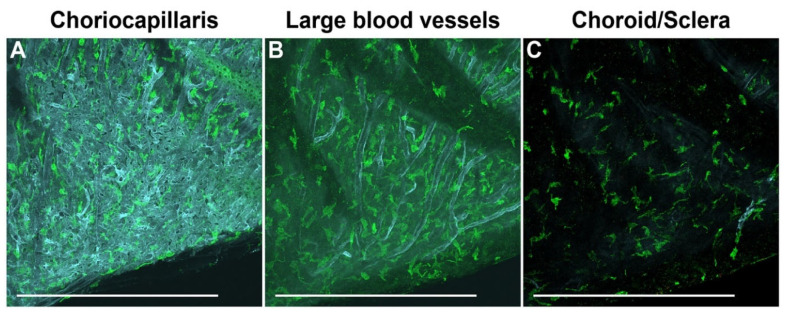
Visualization of macrophages in the choroid of pigmented mice. Macrophages were stained using the anti-ionized calcium binding adaptor molecule 1 (Iba1) antibody. To visualize macrophages with respect to different blood vessel layers, the choroid was co-immunostained with an anti-podocalyxin antibody. Representative images showing macrophages throughout the choroid of C57BL/6J mice, specifically in the choriocapillaris (**A**), large blood vessel layers (**B**), and in the scleral aspect of the choroid (**C**). All representative images were from 6-week-old animals and captured using a confocal microscope. Number of choroids examined = 9. Scale bars: 500 μm.

To visualize macrophages in the mice choroid, we used the anti-ionized calcium-binding adaptor molecule 1 (Iba1) antibody. This marker is used by others to label macrophages in the choroids of humans and albino mice [16,17]. Figure 6 shows representative images of macrophages in the choroid of C57BL/6J mice. When visualized along with blood vessels, macrophages were found in all layers of the choroid, including the choriocapillaris (Figure 6A), large blood vessels (Figure 6B), and the scleral aspect of the choroid (Figure 6C).

### 3.8. Co-Visualization of Blood Vessels, Mast Cells, and Macrophages in the Choroid of Pigmented Mice

**Figure 7:**
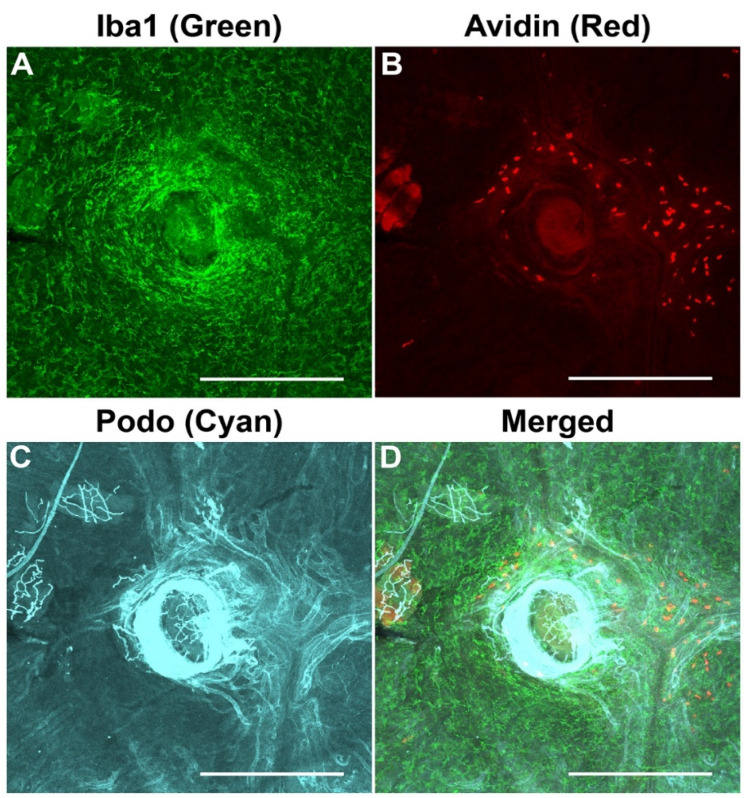
Co-visualization of blood vessels, mast cells, and macrophages in the choroid of pigmented mice. Macrophages, mast cells, and blood vessels in the choroid were labeled with anti-Iba1 antibody, rhodamine-avidin, and anti-podocalyxin, respectively. Representative images from stained and bleached mouse choroid showed robust labeling of macrophages (**A**), mast cells (**B**), and blood vessels (**C**). In addition, merged images (**D**) showed each targeted cell type with respect to other types of cells/blood vessels of the choroid. All representative images were from 6-week-old C57BL/6J animals and captured using confocal microscopy. Number of choroids examined = 9. Scale bars: 500 μm.

We successfully stained the choroid from C57BL/6J mice with podocalyxin and Iba1 antibodies to visualize the vasculature and macrophages, respectively, and with rhodamine-avidin to visualize mast cells (Figure 7A–D). Thus, the method described here allows the simultaneous examination of a number of cell types in the choroid and evaluates potential changes in their interactions, localization, and density.

## 4. Discussion

Utilizing the methods described here revealed several novel findings especially in pigmented mice. (i) Staining with α-SMA facilitated the visualization of an arterial circle around the optic nerve in mice that is similar to the Zinn–Haller arterial circle known to exist in humans and primates. (ii) Staining with podocalyxin facilitated the visualization of the choriocapillaris. Three different structural patterns of choriocapillaris were observed throughout the whole choroid: honeycomb-like, maze-like, and finger-like patterns. The distribution of these patterns was highly variable among strains at the age we examined. (iii) To the best of our knowledge, this is the first report of the visualization of mast cells and macrophages in eyes from pigmented mice. Mast cell distribution and density were found to vary in eyes from different strains and in eyes from different animals of the same strain. (iv) Utilizing the staining and bleaching method described in this report allowed the visualization of the vasculature, mast cells, and macrophages, independently or simultaneously, in the choroid of pigmented mice.

The ocular choroidal tissue contains blood vessels and stroma, the extravascular connective tissue. Choroidal blood vessels are composed of arteries, arterioles, veins, venules, and a capillary bed, the choriocapillaris. Blood vessels in the choroid become smaller as they branch while descending from the sclera border towards the Bruch’s membrane. This branching pattern allows the distinction of three vascular layers: the Haller’s layer of the large vessels close to the sclera; Sattler’s layer of medium size vessels; and the choriocapillaris layer of a highly anastomosed capillary bed next to Bruch’s membrane. The stroma is a connective tissue that encompasses the blood vessels and harbors immune cells, such as mast cells and macrophages, neural tissues, and other types of cells. Choriocapillaris and Bruch’s membrane are fused together via a basement membrane and the intercapillary pillars (also called septa), which is part of the choroidal connective tissue.

The integrity of the choroid is critical for the homeostasis of the RPE and the outer retina. It is well established that the degeneration of the choroid is causally linked to many eye diseases, such as AMD, diabetic choroidopathy, myopia, uveal autoimmune diseases, and retinopathy of prematurity. These diseases cause visual impairments in millions of people each year. However, cellular and molecular pathologic mechanisms responsible for choroidal damage in these diseases are largely unknown. While mouse models have been successfully used to better understand the pathologic mechanisms responsible for human diseases that occur in the retina, their use in diseases related to damage in the choroid have been limited. Investigating the choroid in mice has been challenging because of the lack of simple and reliable methods to visualize blood vessels and inflammatory cells in the choroid. To the best of our knowledge, this is the first detailed report that provides simple methods that enable the visualization of blood vessels, mast cells, and macrophages, independently or simultaneously, in albino and pigmented strains of mice. In this report, we describe in detail the structure of the choroidal vasculature and the distribution of mast cells, and macrophages in three albino (FVBN, CD-1, BALB/cJ) and one pigmented (C57BL/6J) mouse strains.

The choroidal vasculature has been partially visualized in mice using corrosion cast or venous infusion with fluorescent materials [16,26,27]. Lutty’s group reported in an ARVO abstract the use of anti-podocalyxin to stain the choroidal vasculature of pigmented mice [28]. The studies presented here provide a complete detailed description of the mouse choroidal vasculature among several strains. Staining with anti-α-SMA antibody showed that the two LPCAs feed the entire choroidal vasculature and the blood vessels in the iris and the ciliary body. We could not identify short posterior ciliary arteries (SPCAs) in the choroid of mice. SPCAs exist in the choroid of humans. Branches from LPCAs were observed to go around the optic disc forming a vascular circle, such as the Zinn–Haller arterial circle in the choroids of humans and primates. Podocalyxin staining made it easy to visualize the venous system and the choriocapillaris in the choroid. Two venous systems could be found, one is located in the center around the optic disc that seems to drain through the posterior ciliary vein. The second venous system is comprised of four vortex veins located close to the equator on the dorsal, ventral, nasal, and temporal sides of the choroid. Vortex veins drain blood from the iris, periphery of the choroid, and the portion of the posterior choroid close to the equator.

Podocalyxin staining also allowed the detailed visualization of choriocapillaris. Three different patterns in different parts of the choroid could be seen: finger-like projections, regular dense honeycomb, and irregular maze-like patterns. These studies demonstrated similarities and differences in choriocapillaris among different mouse strains, including albino and pigmented mice. Our studies showed that the choroidal vasculature in mice is similar to that in rats, which was studied using the corrosion cast and scanning electron microscope [29]. However, this rat study did not find a vascular circle similar to the Zinn–Haller arterial circle that exists in the choroid of humans and primates, which we showed is present in mice. No lobular structures were found in the choroid of mice, which is similar to the choroid of rats [29]. Choroidal lobuli exist in human and primate eyes [5,30].

Mast cells exist in the choroid of the eye in mammalian species including mice. Staining with avidin allowed the visualization of mast cells in the choroid from different albino and pigmented mice. Assessing the distribution and counting of mast cells revealed many facts about this cell type in the choroid of mice. Mast cells are normally located around the major blood vessels. Mast cell density and distribution in the choroid varied at different levels. It varies in different locations in the same choroid, among individuals from the same strain, and among strains. Visualizing the macrophages revealed their distribution in all layers of the choroid in mice. Collectively, the method described here allows the study of the choroid from albino and pigmented mice at cellular levels, which has the potential of advancing our understanding of the molecular and cellular mechanisms contributing to various ocular pathologies including AMD.

## Data Availability

All the data presented here are included in the manuscript. Further inquiries should be directed to the corresponding author.

## Supplementary Materials

The following are available online at https://www.mdpi.com/article/10.3390/cells11203329/s1, Video S1: 3-D Visualization of the Choroidal Vasculature.
